# Comparative Study of Vitamin D Levels After Metabolic Bariatric Surgery in Women Under or Over 45 Years of Age

**DOI:** 10.1007/s11695-025-08459-3

**Published:** 2026-01-10

**Authors:** Diya Humeida Omer, Khiria Alsaghir, Aye A. Thant, Siba Senapati, Basil J. Ammori, Akheel A. Syed

**Affiliations:** 1 Department of Diabetes, Endocrinology & Obesity Medicine, Salford Royal Hospital,, Northern Care Alliance NHS Foundation Trust, Salford, UK; 2 Department of Bariatric & Oesophago-gastric Surgery, Salford Royal Hospital, Northern Care Alliance NHS Foundation Trust, Salford, UK; 3 School of Health and Society, University of Salford, Salford, UK; 4 Department of Bariatric, General, Gastrointestinal and Hepatobiliary Surgery, Burjeel Hospital, Abu Dhabi, United Arab Emirates; 5 Division of Diabetes, Endocrinology & Gastroenterology, Faculty of Biology, Medicine & Health, University of Manchester, Manchester, UK

**Keywords:** Bariatric surgery, Vitamin D, Calcium, Women, Childbearing age, Parathyroid hormone, Micronutrient deficiency

## Abstract

**Background:**

Vitamin D deficiency is common in people with obesity and can worsen after bariatric surgery. As the reproductive years and post-menopausal status can place additional demands on vitamin D requirements, we studied vitamin D status after bariatric surgery in women under 45 years of age compared with women over 45.

**Methods:**

We conducted an observational cohort study of 305 women undergoing primary bariatric surgery at a university teaching hospital in North West England. Participants were stratified by age into women under 45 years (Wu45, *n* = 123) and over 45 years (Wo45, *n* = 182). Patients were routinely prescribed daily calcium and vitamin D supplementation after bariatric surgery. Serum 25-hydroxyvitamin D, adjusted calcium, parathyroid hormone (PTH) and metabolic parameters were measured preoperatively and at intervals over 24 months postoperatively.

**Results:**

After bariatric surgery, vitamin D levels rose significantly within 4 months but were lower in Wu45 at 12 and 24 months (*p* < 0.05). Adjusted calcium levels declined over time, with Wu45 showing significantly lower levels at 12 and 24 months. PTH levels, initially lower in Wu45, increased and equalized with Wo45’s levels by 12 months.

**Conclusions:**

Women under 45 are at increased risk of vitamin D and calcium deficiencies after bariatric surgery. This may reflect higher physiological demands and variable adherence to supplementation. Patient education and tailored supplementation strategies may be required to prevent long-term micronutrient complications.

## Introduction

Obesity is associated with decreased circulating levels of vitamin D [1, 2]. Reasons for this can include sequestration of fat-soluble vitamin D in adipose tissues, volumetric dilution in larger body mass, and exacerbation of seasonal fluctuation in levels due to reduced outdoor activity and sun exposure. Vitamin D deficiency results in reduced dietary calcium absorption and a compensatory increase in parathyroid hormone (PTH), which stimulates calcium resorption from bones to restore circulating levels. Calcium and vitamin D deficiencies may lead to various health issues, including osteomalacia and osteoporosis and consequent increased risk of fractures, muscle weakness, increased susceptibility to infections and inflammation, mood disturbances, and a close link to insulin resistance [3, 4].

Whilst lifestyle, dietary and behavioral modifications, and anti-obesity medications are widely recommended for obesity management [5, 6], metabolic/bariatric surgery (MBS) remains the most effective treatment for chronic severe obesity. Bariatric surgery leads to significant weight loss and resolution of obesity-related medical, social, and psychological distress [7, 8]. However, all types of MBS could exacerbate pre-existing vitamin D deficiency by reducing dietary intake due to restrictive effect. Gastric and duodenal bypass operations can also aggravate vitamin D deficiency by impeding bile flow and the surface area of vitamin D absorption in the duodenum and jejunum [9].

Calcium and vitamin D status after bariatric surgery is of particular importance in women of childbearing age as pregnancy and lactation place additional demands on nutrient requirements [10]. Calcium metabolism is also influenced by menopause as low estrogen decreases serum calcium levels and the activity of vitamin D in bones and gut [11].

Vitamin D status has been widely studied in people with obesity [2], and before after bariatric surgery [12], including by our group previously [13]. However, whether vitamin D levels in women after bariatric surgery vary by childbearing or menopausal status is not known.

### Aim of the Study

We aimed to study vitamin D status before and after bariatric surgery in women of childbearing age compared with women of non-childbearing age.

## Methods

We performed an observational cohort analysis of prospectively recorded longitudinal data on vitamin D and related parameters in women who underwent bariatric surgery.

### Patients and Setting

We studied women who had undergone primary bariatric surgery based on national guidelines [14, 15] at a university teaching hospital in North West England, with follow-up over 24 months. The study comprised a post-hoc subset analysis of 305 women of our previous study of patients who underwent primary bariatric surgery between March 2012–March 2016 [13]. Follow-up data was available for up to 274 women at 4 months, 245 women at 12 months and 126 women at 24 months. The bariatric surgical procedures were performed laparoscopically as described previously [16]. Following bariatric surgery, patients were routinely recommended oral supplementation with elemental calcium ≥ 1200 mg and cholecalciferol ≥ 800 IU daily on a long-term basis [9]. The patients in the study were categorized into women under 45 years of age (Wu45) or women over 45 years of age (Wo45) as per English census criterion for childbearing age [17]. Data collected included patient demographics, preoperative baseline data and postoperative follow-up data including weight, height, body mass index (BMI; kg/m^2^); and blood tests including total vitamin D (25-hydroxyvitamin D, 25(OH)D), phosphate, alkaline phosphatase (ALP), PTH, albumin and total calcium. The laboratory-recommended reference ranges at the time of the study were used. The calcium level was adjusted for serum albumin < 40 g/L by applying the formula, adjusted calcium (mmol/L) = total calcium (mmol/L) + 0.02 × [40 − albumin (g/L)]. Vitamin D levels were categorized using the thresholds recommended by the National Osteoporosis Society as deficient (25(OH)D < 25 nmol/L; < 10 ng/mL), insufficient (25 to 50 nmol/L; 10 to 20 ng/mL) or sufficient (> 50 nmol/L; > 20 ng/mL) [18].

### Statistical Analysis

We performed descriptive statistics of demographic data with parametric tests (or non-parametric tests for non-normative data), with measures of dispersion as appropriate. To analyze between-subjects differences in continuous variables (namely BMI, vitamin D, calcium and PTH) over time, we performed general linear model (GLM) repeated measures test. Where significant differences were detected, comparative analyses were performed with independent or paired samples *t* tests as appropriate. The Fisher exact test was used to analyze contingency tables of categorical variables. Linear associations were analyzed with the Pearson correlation formula. A two-sided *p* < 0.05 was considered statistically significant.

To cope with missing values because of the retrospective, observational nature of the study, frequencies were reported as valid percentages. Significant results were confirmed by re-analysis after imputing missing data by fully conditional specification. Results were reported on original unimputed data. To correct for multiple testing, the Benjamini-Hochberg adjustment for false discovery rate was applied. Data were analyzed using IBM SPSS Statistics 30.0.0 (IBM Corp, Armonk, NY).

## Results

A total of 305 women were included, comprising 123 (40.3%) women less than 45 years of age (Wu45) and 182 (59.7%) women ≥ 45 years of age (Wo45). Whilst weight was relatively greater in Wu45, there was no difference in BMI between groups; PTH levels were lower in Wu45 but there were non-significant (ns) differences in other baseline characteristics (Table 1).

**Table 1:** Baseline characteristics of all patients and stratified by age

	All, *n* = 305	Age < 45 years, *n* = 123	Age ≥ 45 years, *n* = 182	p*
Age (years)	47.7 (10.6)	37.2 (5.6)	54.7 (6.5)	< 0.001
Weight (kg)	135.4 (23.3)	136.6 (23.9)	129.0 (20.5)	= 0.003
BMI (kg/m^2^)	49.6 (7.4)	50.4 (8.1)	49.2 (6.9)	ns
Vitamin D (nmol/L)^†^	35.9 (25.5)	33.7 (23.2)	37.4 (27.0)	ns
Adjusted calcium (mmol/L)	2.33 (0.10)	2.32 (0.10)	2.34 (0.10)	ns
Phosphate (mmol/L)	1.1 (0.2)	1.1 (0.2)	1.1 (0.2)	ns
Alkaline phosphatase (U/L)	87.3 (29.1)	82.9 (21.9)	90.3 (32.7)	= 0.02
Total protein (g/L)	71.8 (4.3)	72.0 (3.7)	71.7 (4.7)	ns
Albumin (g/L)	43.4 (2.6)	43.5 (2.2)	43.3 (2.9)	ns
PTH (pmol/L)	5.7 (3.3)	5.1 (2.9)	6.1 (3.4)	= 0.03
HbA1c (mmol/mol)	47.8 (16.5)	46.1 (16.5)	48.9 (16.5)	ns
Gastric bypass	186 (61%)	78 (63.4%)	108 (59.3%)	ns
Sleeve gastrectomy	113 (37%)	42 (34.1%)	71 (39.0%)	ns
Gastric banding	6 (2%)	3 (2.4%)	3 (1.6%)	ns

Mean (SD) or count (%). *Independent samples Student t test for continuous variables, Fisher Exact test for categorical variables. ^†^1 nmol/L = 0.4 ng/mL. BMI, body mass index. PTH, parathyroid hormone. ns, non-significant

**Fig. 1:**
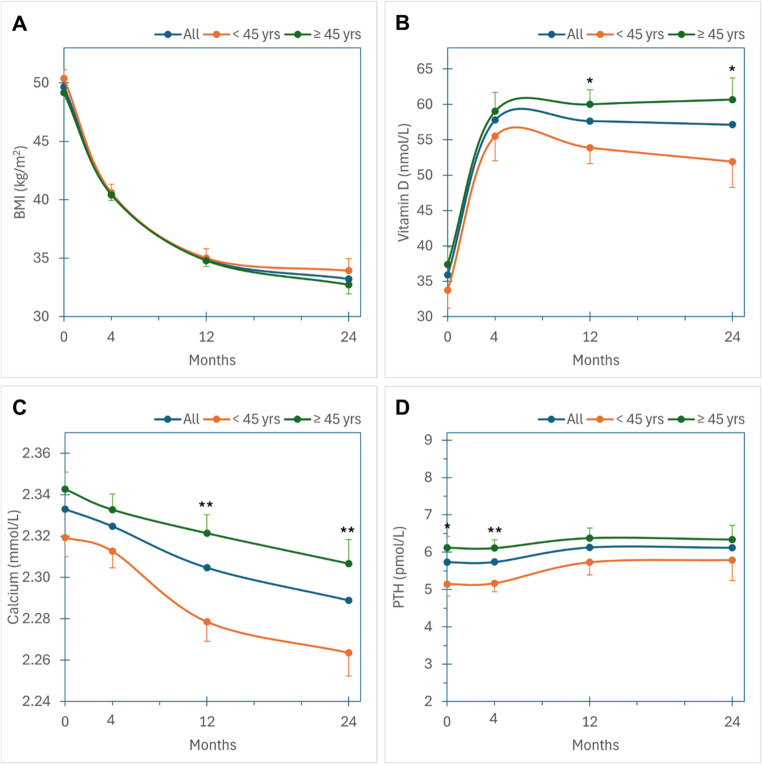
Longitudinal changes over 24 months of follow-up in all women (blue trace), in women under 45 years of age (orange trace) and in women over 45 years of age (green trace). (**A**) Body mass index (BMI). (**B**) Serum vitamin D; 1 nmol/L = 0.4 ng/mL. (**C**) Serum adjusted calcium. (**D**) Parathyroid hormone. Points are mean values; error bars are standard errors. * *p* < 0.05; ** *p* < 0.01

Mean (standard deviation, SD) BMI at baseline was 50.4 (8.1) kg/m^2^ in Wu45 and 49.2 (6.9) kg/m^2^ in Wo45 (ns). Following bariatric surgery, there was significant weight loss with no significant difference in BMI between Wu45 and Wo45 (33.9 vs. 32.7 kg/m^2^) at 24 months (Fig. 1A).

Mean (SD) serum vitamin D levels at baseline were 33.7 (23.2) nmol/L (13.5 (9.3) ng/mL) in Wu45 and 37.4 (27.0) nmol/L (15.0 (10.8) ng/mL) in Wo45 (ns) and increased significantly within 4 months after bariatric surgery, but levels were lower in Wu45 compared with Wo45 at 12 months (53.9 vs. 60.0 nmol/L (21.6 vs. 24 ng/mL), *p* = 0.02) and 24 months (51.9 vs. 60.7 nmol/L (20.8 vs. 24.3 ng/mL, *p* = 0.03) (Fig. 1B). Serum adjusted calcium levels at baseline were 2.32 (0.10) mmol/l in Wu45 and 2.34 (0.10) mmol/L in Wo45 (ns) and decreased over the 2 years of follow-up with levels significantly lower in Wu45 compared with Wo45 at 12 months (2.28 vs. 2.32 mmol/L, *p* = 0.001) and 24 months (2.26 vs. 2.31 mmol/l, *p* = 0.005) (Fig. 1C). PTH levels were stable overall; in Wu45, they were lower at baseline and up to 4 months after MBS but there was no significant difference between groups at 12 and 24 months (Fig. 1D).

**Fig. 2:**
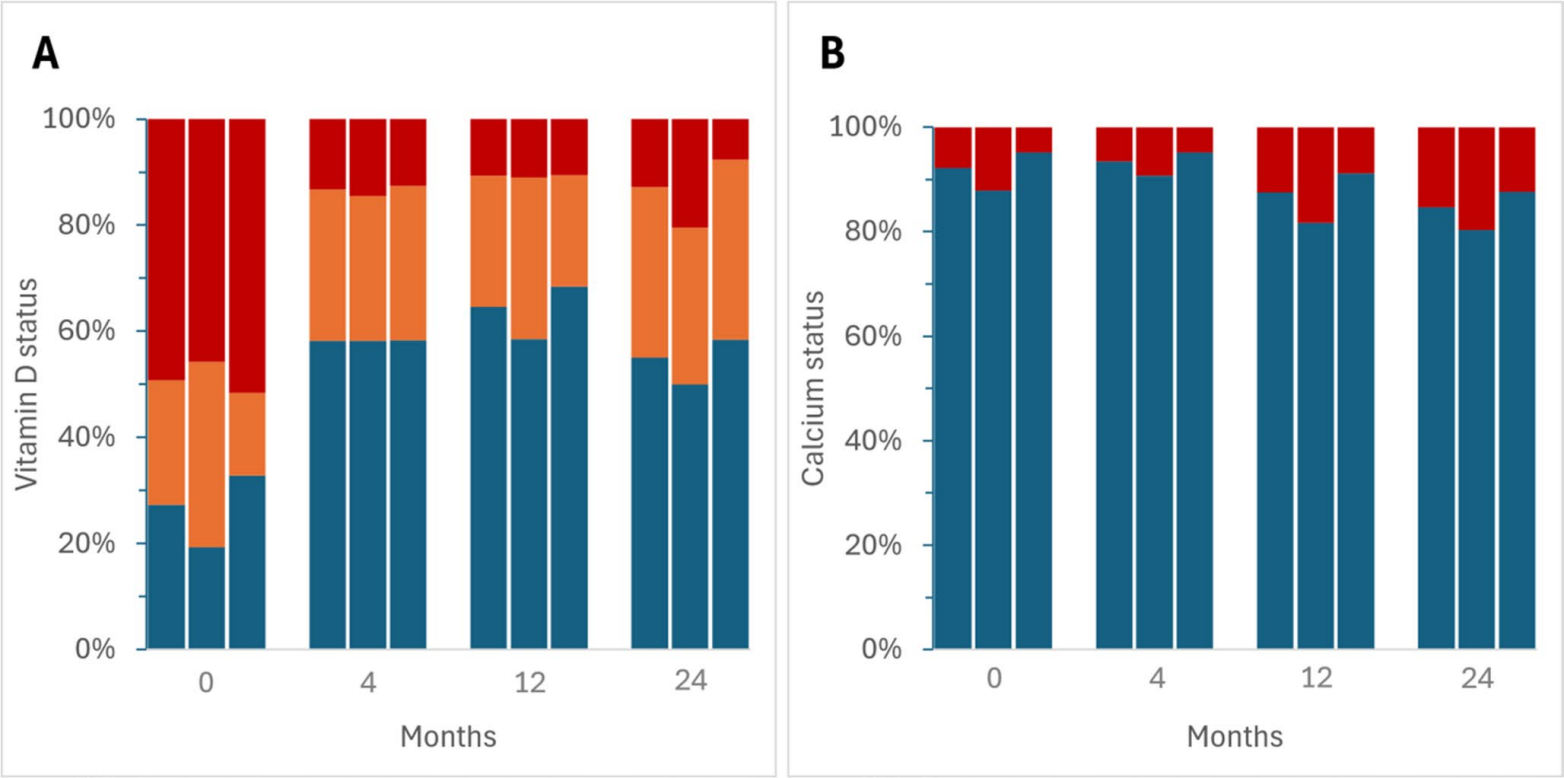
Vitamin D and calcium sufficiency status over 24 months of follow-up in all women (first column in each set), in women under 45 years of age, second column) and in women over 45 years of age, third column). (**A**) Vitamin D status is depicted as sufficient (> 50 nmol/L (> 20 ng/mL), blue stack), insufficient (25 to 50 nmol/L (10 to 20 ng/mL), orange stack) and deficient (< 25 nmol/L (< 10 ng/mL), red stack). (**B**) Adjusted calcium status is depicted as satisfactory (≥ 2.20 mmol/L, blue stack) or low (< 2.20 mmol/L, red stack)

Vitamin D levels in all women were in the sufficient range in 26.9% at baseline, 58.2% at 4 months, 64.7% at 12 months and 55.0% at 24 months (Fig. 2A). At baseline, Wu45 had lower vitamin D sufficiency rate compared with Wo45 (19.3% vs. 32.8%, *p* = 0.038). Vitamin D sufficiency rates improved and were similar in both groups by 4 months (58.2% vs. 58.3%, ns), improved further in Wo45 by 12 months (58.5% vs. 68.4%, ns), and declined in both groups with a greater decline in Wu45 by 24 months (50.0% vs. 58.5%, ns). Adjusted calcium levels in all women were in the satisfactory range in 92.2% at baseline, 93.4% at 4 months, 87.5% at 12 months and 84.7% at 24 months (Fig. 2B). At baseline, Wu45 had 'satisfactory calcium' rate lower compared with Wo45 (87.8% vs. 95.2%, *p* = 0.040). Calcium sufficiency rates were similar in both groups by 4 months (90.7% vs. 95.2%, ns) but declined more in Wu45 by 12 months (81.7% vs. 91.2%, *p* = 0.044) and 24 months (80.4% vs. 87.7%, ns).

There were no significant differences or time trends in serum phosphate levels between groups. Alkaline phosphatase was lower in Wu45 compared with Wo45 at all timepoints including baseline (82.9 vs. 90.3 U/L, *p* = 0.02), 4 months (83.3 vs. 95.5 U/L, *p* = 0.002), 12 months (78.7 vs. 92.4 U/L, *p* < 0.001) and 24 months (73.1 vs. 86.6 U/L, *p* = 0.006).

## Discussion

In a two-year longitudinal study, we assessed the impact of bariatric surgery on levels of vitamin D, serum calcium and PTH in women categorized by reproductive age. We found that vitamin D levels prior to bariatric surgery were similar in women irrespective of reproductive age group. Vitamin D levels increased after surgery overall, which may be attributed to several factors including adherence to postoperative vitamin D supplementation, release of sequestered vitamin D from adipose tissues with rapid weight loss especially in the initial months after surgery, and improved outdoor physical activity and sun exposure.

Vitamin D levels were stable over two years of follow-up in women over 45 but were significantly lower in women under 45 by 12 and 24 months. This could be attributed to irregular adherence to the prescribed supplements in women under 45 due to less concern about the risk of post-menopausal osteoporosis [19–21]. It has been reported that younger adults were found to be less adherent, with higher reports of unintentional medication nonadherence, than older adults [22]. A review of patient adherence to multivitamin supplementation after bariatric surgery also found younger (< 40 years of age) and middle-aged patients (40–54 years) had lower adherence compared with older patients (> 55 years) [23].

Women under 45 also have higher physiological demands for macro- and micro-nutrients to meet requirements during the menstrual cycle, pregnancy, delivery, and lactation [10, 24, 25]. Higher demands for calcium during active menstruation and fertility, mediated through anabolic effects of estrogen, contribute to this picture [26, 27]. Moreover, women under 45 may be prone to weight regain, and re-sequestration of vitamin D in adipose tissue, during periods of pregnancy and puerperium [28, 29].

Serum adjusted calcium levels decreased over the 2-year follow-up period in both groups, with levels being significantly lower in Wu45 compared with Wo45 at 12 and 24 months. This could be attributed to reduced dietary intake due to the physical restriction that applies to all types of bariatric surgery and impaired absorption due to bypassing of calcium absorption sites in the duodenum and jejunum (gastric and duodenal bypass operations), in addition to reduced gastric acid secretion (partly due to the use of proton pump inhibitors) that is necessary for digestion and absorption [30, 31]. Vitamin D insufficiency before or after surgery may exacerbate these lower levels of calcium [32–34].

PTH levels were stable overall; in Wu45, they were lower at baseline and up to 4 months after bariatric surgery compared with Wo45, but there was no significant difference between groups at 12 and 24 months. However, there was a trend of rising PTH levels over the two years of follow-up, particularly in women under 45. This could reflect a compensatory rise in PTH due to the tendency to calcium and/or vitamin D insufficiency over time which was clearly evident in this cohort. Other factors influencing the PTH trends could include increased chronic bone resorption activity in general, and in women over 45 in particular due to estrogen deficiency that may lead to paradoxical stimulation of PTH in the longer term after surgery. Eventually both groups will reach equilibrium of PTH status after a period of adaptation as they balance their needs through diet and supplementation. However, perturbations of PTH status, as a surrogate marker of bone turnover, could be indicative of long-term bone health.

Alkaline phosphatase is recognized to increase with age, particularly in women after the menopause due to increased bone turnover [35]. This was reflected in higher average alkaline phosphatase levels in women over 45 years of age in our study cohort.

For longer-term monitoring of serum vitamin D levels [9], annual blood tests may overlook timing of vitamin D measurements which can be lower in winter than in summer months [36]. Whilst oral supplementation with cholecalciferol ≥ 800 IU daily is the minimum standard for persons who have undergone bariatric surgery, higher doses are required, particularly for patients with insufficiency/deficiency, to reach serum vitamin D levels ≥ 75nmol/L (30 ng/mL) [9]. Maintenance doses of between 2000 and 4000 IU of oral cholecalciferol per day may be required following sleeve gastrectomy and gastric bypass operations and higher following malabsorptive procedures such as biliopancreatic diversion/duodenal switch [9].

### Strengths and Limitations

As a large series of over 300 participants with prospectively accumulated data on calcium, vitamin D and PTH levels longitudinally over 2 years of follow-up, we show statistically significant and clinically meaningful results. Whilst a single-center study, which may limit its diversity and generalizability, our findings are broadly in keeping with previous studies of calcium and vitamin D status after bariatric surgery [37]. Furthermore, as the first comprehensive study to examine differences in calcium and vitamin D status in women under 45 compared with women over 45, we provide important insights with implications for not only general health and wellbeing but also maternal and post-reproductive health.

However, classification of women under and over the age of 45, by lacking biological accuracy, may be misleading and includes heterogeneous and diverse women at different fertility and menopausal statuses without referring to clinical manifestations or hormonal markers, which were beyond the scope of this study. We did not have information on pregnancy events post-bariatric surgery which could also influence calcium and vitamin D status. However, it is standard practice in our center to advise women to avoid pregnancy for at least 12 months, preferably 24 months, after bariatric surgery, consistent with American College of Obstetricians and Gynecologists (ACOG) guidelines [38]. Additionally, being a retrospective cohort study may be associated with selection bias and the loss of some data, mitigated by statistical techniques for missing data. The study findings may be influenced by other factors, such as dietary behaviors, and varying levels of sun exposure in the region. Whilst a minimum intake of cholecalciferol 800 units daily was recommended, variable (higher) doses of supplementation and variable degrees of adherence, which were beyond the scope of this study to ascertain, could have also affected the study results. As a study of routine NHS clinical practice, we also did not have measurements of advanced bone turnover markers or bone mineral density. Despite these limitations, our study provides meaningful insights for optimal clinical management of calcium and vitamin D status, and potentially long-term bone health, in women of all ages.

## Future Research

Comprehensive assessment of bone health in persons who have undergone bariatric surgery is under-researched. Future work should include longitudinal measurements of bone mineral density by dual energy x-ray absorptiometry (DEXA) or quantitative computed tomography (CT) or magnetic resonance imaging (MRI), biochemical markers of bone turnover.

## Conclusion

Bariatric surgery can affect the absorption of micronutrients such as calcium and vitamin D, and women under 45 are at greater risk of insufficiencies of these micronutrients postoperatively. For optimal long-term outcomes from bariatric surgery, patient education and tailored calcium and vitamin D supplementation are essential.

## Data Availability

The datasets generated or analysed during the current study are available from the corresponding author upon reasonable request, subject to institutional and national data protection regulations.
